# Caregiver satisfaction and its associated factors in pediatric wards of Jimma University Medical Center, Southwest Ethiopia

**DOI:** 10.1186/s12913-022-08459-4

**Published:** 2022-08-19

**Authors:** Mohammed Beshir, Tsion Tilahun, Diriba Fufa Hordofa, Gersam Abera, Workineh Tesfaye, Kumale Tolesa Daba, Netsanet Workineh, Samson Nadew Woldeyesus, Tilahun Fufa Debela, Elias Ali Yesuf

**Affiliations:** 1 School of Medicine, Institute of Health, Jimma University, Jimma, Ethiopia; 2 Public Health Faculty, Institute of Health, Jimma University, Jimma, Ethiopia

**Keywords:** Caregiver, Satisfaction, Pediatric, And Jimma University

## Abstract

**Background:**

Measuring the caregivers' satisfaction is vital for the effectiveness of childcare. Children admitted to pediatric wards require special hospital situations, a bespoke approach from the medical team, and the participation of caregivers. It is important to give attention to the caregivers while planning for child services. The purpose of this study was to assess the caregiver’s satisfaction with the services provided in pediatric wards of Jimma University Medical Center and identify its associated factors.

**Methods:**

Facility-based cross-sectional study design was done at Jimma University Medical Center. Participants in the study were primary caregivers who spent two or more days in the pediatric ward with their admitted children. Data were collected from 400 selected caregivers. A consecutive sampling method was employed. Principal component analysis was done for all Likert scale instruments to extract factor (s) representing each of the scales and to have factor scores. Variables with the Likert scale were treated as continuous after principal component analysis was employed. Using these factor scores, a multiple linear regression analysis was carried out to identify factors associated with caregivers' satisfaction with service in pediatric wards. A significance level of less than 0.05 was used in the final model to determine statistical significance.

**Result:**

This study showed that caregivers' satisfaction with the services in the pediatric ward was 68%. Level of education (β = -0.24, (95% CI; -.411, -.070)), availability of basic facilities (β = 0.163, (95% CI; .063, .263)), patience to listen to patients' problem (β = 0.431, (95% CI; .324, .537, staff communication with client (β = 0.163, (95% CI; -4.839, -1.610)), availability of laboratory tests and its service (β = -0.964, (95% CI; -1.435, -.493)), availability of drug, imaging and service at radiology (β = 2.907, (95% CI; 1.470, 4.344)) and availability of pathology and its service (β = 1.869, (95% CI; .993, 2.746)) were significant factors associated with caregivers satisfaction with health service in pediatrics ward.

**Conclusion:**

Caregivers were moderately satisfied. Education level, availability of basic facilities communication with client, and the availability of laboratory tests and drugs were factors that significantly associated with caregiver satisfaction. Hospital should avail laboratory tests and drugs in order to maintain high levels of caregiver satisfaction.

## Background

Child mortality remains the leading public health problem around the globe. Four-fifths of all deaths among children and young adults are caused by under-five mortality [1]. The problem is especially worse in developing nations. A high child mortality rate is a symptom of an unfavorable socioeconomic condition during childhood [2] and demonstrates how well a nation's health care system is functioning. From 1990 to 2020, there was a significant drop in child mortality, down from 12.6 million to 5 million [3]. Child mortality is fourteen times more likely in Sub-Saharan countries than it is in Europe and Northern America [4]. The mortality rate for children under five is 59 per 1000 live births in 2019 [5]. Preterm birth complications, birth asphyxia/trauma, pneumonia, congenital anomalies, diarrhea, and malaria are the main causes of death in children under five years [6].

Patients admitted to the pediatric ward require a particular hospital context, a specialized medical team approach, and caregiver involvement [7]. Hence, when planning to provide quality services, it is crucial to focus on the caregivers [8]. One crucial result of care is satisfaction [9]. Caregiver satisfaction is a crucial metric for assessing the quality of medical care [10]. It affects patient adherence, clinical outcomes, and malpractice claims [11]. Pediatric inpatient treatment is not complete without the support and satisfaction of the caregivers [12]. Understanding and fulfilling the expectations of the caregivers requires assessing their satisfaction [13].

Satisfaction is complex concept and is an important factor in increasing patient retention and honesty [14]. It is influenced by a variety of factors, including societal norms, the context in which health care is delivered, the need for and value of caregivers, and personal experiences [13, 15, 16]. Expectations, educational level, health, ability to receive medical care, knowledge, and other psychological factors all play a role [17–20].

To provide patient-centered treatment and guarantee patient adherence, it is essential to involve families or caregivers in decisions regarding child care [21]. The perception of the caregivers the most important factor in shaping service uses [22]. Customers did not use the service if they were disappointed with some aspects [13, 15, 23]. Knowing the consumer's perspective on a single service and determining their level of satisfaction with the services offered is critical for improving the services [10]. In this regard, there is a scarcity of information on caregiver satisfaction, particularly in the study areas. Jimma University Medical Center is the only referral hospital in Southwest Ethiopia. It serves as a referral point for more than 15 million people in various regions and neighboring countries. The hospital strives to modify its services on a regular basis and to make patient-centered care. Hence, the purpose of this study was to assess caregiver satisfaction with pediatric ward care and identify its associated factors. The findings of this study are important for identifying caregiver satisfaction and improving service quality in pediatric departments. Furthermore, the findings of this analysis assist health planners and implementers in taking the aforementioned factors into account in order to improve service utilization.

## Methods

### Study design, setting and period

An institutional-based cross-sectional study design was conducted at Jimma University Medical Center (JUMC) from June 02, 2020, to August 07, 2020. Jimma University Medical Center is one of the oldest hospitals in Ethiopia and it is the only teaching hospital in Southwest Ethiopia. It is found in Jimma town which is located 352 km from Addis Ababa, the capital. The medical center serves the catchment population of 15 million people from a diverse population from three regional states; namely, Oromia regional state, South Nation Nationalities and people’s regional state, and Gambella people regional state.

### Study population

All caregivers who visited the medical center as primary caregivers of the admitted children in the pediatric ward were considered as the source population. All randomly selected caregivers who stayed in the pediatric ward for two or more days in the ward with the admitted children participated in the study. Caregivers who were unable to speak, and hear were excluded from the study.

### Sample size determination and sampling technique

The sample size was determined using a single population proportion formula by considering a 50% proportion of caregivers' satisfaction with the services of pediatric wards, a 95% confidence level, and a 0.05 margin of error. Consequently, the calculated sample size became 384. By adding a 10% non-response rate, the final sample used to collect data was 422. To select individual study participants, a consecutive sampling method was employed. The minimum day of stay with the admitted child was two days. If the mother and father of the child were in the ward priority was given to the mother. If caregivers were other than the mother and father of the child, the primary caregiver who stayed more with the children was interviewed.

### Data collection procedure

Data were collected through face-to-face interviews using structured questionnaires. Data collection instrument was developed after reviewing related relevant literatures [10, 12, 16, 17, 19, 24]. The question mainly consisted of close-ended questions addressing socio-demographic characteristics of respondents, client-provider interaction, hospital-related factors, and clinical and admission factors. The questionnaire was first prepared in English and translated into local languages (Afan Oromo and Amharic) and retranslated back into English to ensure its consistency. The pretest was conducted in Shenen Gibe Hospital; outside the study area on 5% of the sample size (*n* = 21). Five trained health professionals who speak the local language and work outside the study hospital collected the data. Data collectors were trained for one day to be familiar with the data collection tool. Editing and sorting of the questionnaires were done to determine the completeness and consistency of data every day at the time of data collection.

### Measurements

#### Caregiver’s satisfaction

Caregiver’s satisfaction was assessed using 10 items on a five-point Likert scale ranging from very dissatisfied [1] to very satisfied [5]. These 10 items were based on the following questions: satisfaction with the communication of health providers, their patience to listen to patient’s problems, satisfaction with information provided, and language used by service providers and respect of health workers. Principal Component Analysis (PCA) was conducted to extract factor/s represent/s and one factor with an eigenvalue greater than one was extracted. The prevalence of the caregiver's satisfaction in this study was calculated by using the percent mean formula: the ratio of actual value minus potential minimum to potential maximum minus potential minimum [25, 26]. If the prevalence is (≥ 80), it is affirmed that there was good satisfaction in the overall aspect of care in the hospital. Moderate (not too bad), when equals to 60% to 80% and considered as low satisfaction if less than 60% [25].

### Client provider interaction

Client-provider interaction was assessed using 13 items on a five-point Likert scale ranging from very dissatisfied [1] to very satisfied [5]. To extract the underlying factor of the client provider interaction, principal component analysis (PCA) was done and one meaningful factor with an eigenvalue greater than one was extracted. During the analysis, the scale was reduced into one item (client-provider interaction satisfaction score) with the Eigenvalue greater than one. The extracted item explained 77.717% of the overall variance and was used as a continuous variable in further analysis.

### Satisfaction with hospital settings

It was assessed using 5 items on five points Likert scale ranging from very dissatisfied [1] to very satisfied [5]. Also, principal component analysis (PCA) was conducted and one factor with an eigenvalue greater than one was extracted. The extracted item explained 76.32% of the overall variance and was used as a continuous variable in further analysis.

### Satisfaction with basic facilities in the hospital

Satisfaction with the basic facility was also assessed using 5 items on five points Likert scale ranging from very dissatisfied [1] to very satisfied [5]. These 5 items were based on the following questions: the cleanliness/neatness of the ward during your stay, suitability of food service, easy accessibility of pipe water, bathroom, and cleanliness of latrine. Principal Component Analysis (PCA) was conducted to extract the underlying factors (components) of the satisfaction with basic facilities scale, and one factor with an eigenvalue greater than one was extracted. The extracted item explained 89.767% of the overall variance and was used as a continuous variable in further analysis.

### Satisfaction with pharmaceutical and diagnostic services

It was assessed using 8 items on five points Likert scale ranging from very dissatisfied [1] to very satisfied [5]. These 8 items were based on the following questions: these services are the availability of laboratory tests and services, availability of drugs in the inpatient pharmacy, availability of imaging modalities, and availability of pathology services in the hospital. Principal Component Analysis (PCA) was conducted and one meaningful factor with an eigenvalue greater than one was extracted. The extracted item explained 94.849% of the overall variance and was used as a continuous variable in further analysis (Table 1).

**Table 1:** The results of reliability coefficient (Cronbach's Alpha) and KMO and Bartlett's Test of composite variable, Jimma, Ethiopia, 2021

Variables	Cronbach's Alpha	KMO (*P* = < 0.001)
Caregiver’s Satisfaction	0.853	0.750
Client Provider Interaction	0.82	0.854
Satisfaction with hospital settings	0.781	0.822
Satisfaction with basic facilities in the hospital	0.844	0.694
Satisfaction with pharmaceutical and diagnostic services	0.744	0.546

### Data analysis

Data were cleaned and entered into EpiData software version 3.1 and then exported to SPSS version 21 for analysis. For the socio-demographic characteristics, descriptive analysis was done. Principal component analysis was employed for all Likert scale instruments to extract factor (s) representing each of the scales and have factor scores, which facilitate treatment of the variables as a continuous during the analysis [27]. Multiple linear regressions were done to identify the determinants of the caregiver's satisfaction. Significance level of 0.05 was used as a cut of point. Throughout the principal component analysis, varimax rotation and eigenvalue of one and above was used. Factors with Cronbach’s alpha value above 0.7 were used in the succeeding analysis. Some factors were renamed in case the scale had above one factor extracted [25].

## Results

### Socio-demographic characteristics of the respondent

Out of 422 populations included in the sample, a total of 400 respondents participated in our study yielding a response rate of 95%. Of these, 213 (53.3%) respondents were female and 327 (81.8%) were Oromo in ethnicity. One hundred forty (35%) of respondents were in the age of 25–29 years with the mean (± SD) age of 30.26(± 7.41) years. Three fourth (75.8%) of the study participants were Muslim in religion while 54 (13.5%) were Orthodox religious followers. More than one-fifth (21.76%) of study participants were among the poorest. Of the respondents, almost all (96%) of them were married. One hundred forty-six (36.5%) of respondents were unable to read and write. More than half (53.8%) of respondents were farmers in occupation. Nearly three fourth (72.3%) of study participants were self-sponsor. Among the study participants, 326 (81.5%) of them visited the pediatric ward as new patients. Three hundred twenty-nine (82.3%) of respondents were came to the medical center by referral from other health institutions (Table 2).

**Table 2:** Socio-demographic characteristics of caregivers in pediatric ward of Jimma Medical Center, Jimma, Ethiopia, 2021

Characteristics		Frequency	Percentage
Sex	Male	187	46.7
	Female	213	53.3
Age of caregivers in year	15–19	10	2.5
	20–24	67	16.8
	25–29	140	35.0
	30–34	72	18.0
	35–39	62	15.5
	40–44	33	8.3
	> = 45	16	4.0
Ethnicity	Oromo	327	81.8
	Amhara	37	9.3
	Kafficho	34	8.6
	Gurage	2	0.5
Religion	Muslim	303	75.8
	Orthodox	54	13.5
	Protestant	43	10.8
Marital status	Married	384	96.0
	Single	7	1.8
	Divorced	8	2.0
	Widowed	1	.3
Educational status	Can’t read and write	146	36.5
	Grade 1–6	109	27.3
	Grade 7–12	84	21.0
	Certificate	13	3.3
	Diploma	31	7.8
	Degree	16	4.0
	Master and Above	1	.3
Occupation	Farmer	215	53.8
	Merchant	67	16.8
	Daily Laborer	47	11.8
	Government Employed	56	14.0
	House Wife	11	2.8
Service fee	By Insurance	80	20.0
	Self-Sponsor	289	72.3
	Fee Waived by Woreda	29	7.3
	Free Service by Hospital	2	.6
Visit	New	326	81.5
	Repeat	73	18.3
Source of referral	Self-Referred	71	17.8
	Referred by the health institution	329	82.3

### Patient satisfaction with Hospital setting related

Of the respondents, 253 (63.2%) of them were dissatisfied with the easy accessibility of the location of the emergency department in the Hospital. Two hundred seventy-one (67.7%) of respondents were satisfied with the cleanliness and comfort of the waiting area. Similarly, 305 (76.4%) of the study participants were dissatisfied with the waiting time before entering the physician's room and 273 (68.2%) respondents were satisfied with the location and easily accessible to the ward. Two hundred ninety (72.5%) of study participants were dissatisfied with the Suitability/comfort of the ward during their stay (Table 3).

**Table 3:** Patient satisfaction with Hospital setting of pediatric ward in Jimma Medical Center, Jimma, Ethiopia, 2021

Characteristics		Frequency	Percent
Easily accessibility of the location of the emergency department in the Hospital	Satisfied	52	13
	Neutral	95	23.8
	Dissatisfied	253	63.2
How you were satisfied with the cleanliness and comfort of the waiting area?	Satisfied	271	67.7
	Neutral	94	23.5
	Dissatisfied	35	8.8
How were you satisfied with the waiting time before entering the physician's room?	Satisfied	29	7.3
	Neutral	65	16.3
	Dissatisfied	305	76.4
How are you satisfied with the location of the ward and easily accessible to visitors in the hospital	Satisfied	273	68.2
	Neutral	71	17.8
	Dissatisfied	56	14
How were you satisfied with the Suitability/comfort of the ward during your stay?	Satisfied	45	11.2
	Neutral	65	16.3
	Dissatisfied	290	72.5

### Patient satisfaction with general facilities in the hospital and healthcare providers

Among the respondents who participated in the study, 267 (66.7%) of them were satisfied with the cleanliness/neatness of the ward during their stay and 191 (47.8%) of the respondents were dissatisfied with the suitability of the food service provided by the hospital. Two hundred three (51%) of the study participants were dissatisfied with the easy accessibility of pipe water and 216 (54%) of respondents were also dissatisfied with the easily accessibility & suitability bathroom. One hundred eighty-nine (47.2%) of respondents were satisfied with the easily accessibility and cleanliness of the latrine. Two hundred seventy-nine (69.8%) of respondents were satisfied with the frontline health care providers' availability and immediately arrival when needed. Of the study participants, 277 (69.2%) of respondents were dissatisfied with the availability of assigned nurses when needed. Two hundred fifty six (64%) of study participants were dissatisfied with the doctors' availability when needed. Majorities (69.2%) of respondents were satisfied with the communication skill of the Doctors but, 302(75.5%) of study participants were dissatisfied with the communication skill of the nurses. Among the study participants, 260 (65%) of them were satisfied with the communication skill of supportive staff. One hundred thirty seven (34.2%) of respondents were satisfied with the health professionals' patience to listen to their problems. Majority (62.2%) of respondents were satisfied with the information given to them. Two hundred fifty nine (64.7%) of study participants were satisfied with the language health professionals used to communicate with them. Three-fourth (74.5%) of respondents were satisfied with the respect from doctors. Two hundred eighty four (71%) of study participants were satisfied with the respect from nurses. Two hundred sixty (65.3%) of respondents were dissatisfied with the respect shown by supportive staff. One-fourth 98(25.8%) of study participants responded there is discrimination by health professionals during the service delivery (Table 4).

**Table 4:** Patient satisfaction with general facilities in pediatric ward of Jimma Medical Center, Jimma, Ethiopia, 2021

**Variable**		**Frequency**	**Percentage**
How were you satisfied with the cleanliness/neatness of the ward during your stay	Satisfied	267	66.7
	Neutral	91	22.8
	Dissatisfied	42	10.5
How were you satisfied with the Suitability of the food service provided by the hospital?	Satisfied	49	12.2
	Neutral	160	40
	Dissatisfied	191	47.8
How were you satisfied with the easy accessibility of pipe water?	Satisfied	104	26
	Neutral	92	23
	Dissatisfied	203	51
How were you satisfied with the easy accessibility & suitability of the bathroom?	Satisfied	109	27.2
	Neutral	75	18.8
	Dissatisfied	216	54
How were you satisfied with the easily accessible and cleanliness of the Latrine?	Satisfied	189	47.2
	Neutral	98	24.5
	Dissatisfied	113	28.3
**Patients' satisfaction with the attitude and behavior of the healthcare providers**			
How were you satisfied with the frontline health care providers’ availability immediately when you arrived?	Satisfied	279	69.8
	Neutral	96	24
	Dissatisfied	25	6.2
How were you satisfied with the availability of assigned nurses when needed?	Satisfied	96	24
	Neutral	27	6.8
	Dissatisfied	277	69.2
How were you satisfied with the doctors’ availability when needed?	Satisfied	117	29.2
	Neutral	27	6.8
	Dissatisfied	256	64
How were you satisfied with the Communication skill of the doctors with you?	Satisfied	277	69.2
	Neutral	95	23
	Dissatisfied	28	7
How were you satisfied with the Communication skill of the nurses with you?	Satisfied	61	15.2
	Neutral	37	9.3
	Dissatisfied	302	75.5
How were you satisfied with the Communication skill of the supportive hospital staff with you?	Satisfied	260	65
	Neutral	72	18
	Dissatisfied	68	17
How you were satisfied with the health professional's patience to listen to the patient's problems?	Satisfied	137	34.2
	Neutral	135	33
	Dissatisfied	28	7
How were you satisfied with the information given to the patients' families about the child's problem or disease?	Satisfied	249	62.2
	Neutral	87	21.8
	Dissatisfied	64	16
How were you satisfied with the language that the health professionals used to communicate with you? Was it simple/ understandable?	Satisfied	259	64.7
	Neutral	87	21.8
	Dissatisfied	54	13.5
How were you satisfied with how Doctors showed respect for you?	Satisfied	298	74.5
	Neutral	75	18.8
	Dissatisfied	27	6.7
How were you satisfied with how the Nurses showed respect for you?	Satisfied	284	71.1
	Neutral	78	19.6
	Dissatisfied	37	9.3
How were you satisfied with the respect shown by the Supportive staff for you?	Satisfied	72	18.1
	Neutral	66	16.6
	Dissatisfied	260	65.3
Is there any discrimination by health professionals during service delivery in hospital	No	180	47.2
	I don’t know	103	27
	Yes	98	25.8

### Satisfaction with pharmaceutical and diagnostic services

More than half (54.5%) of respondents were satisfied with the availability of laboratory tests. One hundred forty seven (38.5%) of the study participants were satisfied with the service provided in the laboratory unit. One hundred twenty one (40.4%) of respondents were dissatisfied with the availability of drugs and 121 (31.8%) of them dissatisfied with the service in the pharmacy units. Two-thirds (66.8%) of the study participants were satisfied with the availability of imaging modalities and 157(59.7%) of them were satisfied with the service in the radiology department. Among the respondents, 99(66.9%) and 81 (65.3%) of them were satisfied with the availability of pathology and services provided in the department. Two hundred sixty six (66.8%) of respondents were satisfied with the cost of the services (Table 5).

**Table 5:** Satisfaction of caregivers with other services in pediatric ward of Jimma Medical Center, Jimma, Ethiopia, 2021

Variables		Frequency	Percent
Availability of laboratory tests in the hospital	Satisfied	218	54.5
	Neutral	58	14.5
	Dissatisfied	124	31
Satisfaction with the service provided in the laboratory services	Satisfied	147	38.5
	Neutral	115	30.1
	Dissatisfied	120	31.4
Availability of drugs in the inpatient pharmacy	Satisfied	122	40.6
	Neutral	57	19
	Dissatisfied	121	40.4
Satisfaction with the service provided in the pharmacy	Satisfied	145	38.2
	Neutral	114	30
	Dissatisfied	121	31.8
Availability of imaging modalities (x-ray, CT scan, Ultrasound)	Satisfied	192	66.8
	Neutral	71	17.8
	Dissatisfied	24	8.4
Satisfaction of service provided by trained staff at the radiology department	Satisfied	157	59.2
	Neutral	84	31.7
	Dissatisfied	24	9.1
Availability of pathology services	Satisfied	99	66.9
	Neutral	34	23
	Dissatisfied	15	10.1
Satisfaction with the service provided in the department of pathology	Satisfied	81	65.3
	Neutral	27	21.7
	Dissatisfied	16	13
**Overall satisfaction with services**			
How are you satisfied with the Cost of the service in the hospital?	Satisfied	266	66.8
	Neutral	99	24.9
	Dissatisfied	33	8.3
Overall satisfaction service of the hospital	Satisfied	302	76
	Neutral	76	19
	Dissatisfied	19	5

### Predictors of the level of caregiver's satisfaction

All predictors of satisfaction with p-values less than 0.25 were entered into a final regression model, and the final predictors of Caregiver satisfaction were identified. The model explains about 35.8% (R square = 0.358) of the variance in job satisfaction. Accordingly, level of education (*p* = 0.006), basic facilities in the hospital (*p* = 0.001), attitude and behavior of health care providers (*p* =  < 0.001), staff communication with the client (*p* =  < 0.001), availability of laboratory tests and their service (*p* =  < 0.001), availability of the drug, imaging, and service at radiology (*p* =  < 0.001) and availability of pathology and its service (*p* =  < 0.001) were appeared to be statistically significant that affect the care taker’s satisfaction.

Holding other variables constant, the satisfaction score of can’t read and write caregivers who visited the pediatric ward of the medical center had a 0.24 unit decrease as compared to those who had ever attended school (β = -0.24, (95% CI; -0.411, -0.070)). This study identified that as the availability of basic facilities in the hospital increased by one unit, caregivers' satisfaction scores increased by 0.163 units (β = 0.163, (95% CI; 0.063, 0.263)). As the patient satisfaction with the health professional’s patience to listen to patients’ problems increased by one unit, the satisfaction score increased by 0.431 unit holding other variables constant (β = 0.431, (95% CI; 0.324, 0.537)). Caregivers who were dissatisfied with the staff communication had an average decrease of 3.225 units in their satisfaction score (β = 0.163, (95% CI; -4.839, -1.610)). Holding other variables constant, as the availability of laboratory tests and services decreased by one unit, caregivers' satisfaction decreased by 0.964 units (β = -0.964, (95% CI; -1.435, -0.493)). As the availability of drugs, imaging, and services in the radiology room increased by one unit, the Caregiver's satisfaction score increased by 2.907 units holding other variables constant (β = 2.907, (95% CI; 1.470, 4.344)). Holding other variables constant, as the availability of pathology services in the hospital and service in the pathology department increased by one unit, caregivers' satisfaction increased by 1.869 units (β = 1.869, (95% CI; 0.993, 2.746)) (Table 6).

**Table 6:** Factors associated with caregiver’s satisfaction in pediatric service of Jimma Medical Center, Jimma, Ethiopia, 2021

Variable		Unstandardized Coefficients	Standardized Coefficients	Sig	95% Confidence Interval for B	
		**B**	**Beta**		**Lower Bound**	**Upper Bound**
(Constant)		.091		.078	-.010	.192
Sex	Female	1				
	Male	.084	.042	.404	-.113	.281
Education level	Literate	1				
	Can’t read and write	-.240	-.116	.006	-.411	-.070
Hospital related factors		-.042	-.042	.435	-.146	.063
Availability of basic facilities		.163	.163	.001	.063	.263
Patience to listen to client’s problem		.431	.431	.000	.324	.537
Satisfaction with staff communication		-3.225	-3.213	.000	-4.839	-1.610
Availability of laboratory tests and service		-.964	-.961	.000	-1.435	-.493
Availability of drugs, imaging, and service in radiology		2.907	2.886	.000	1.470	4.344
Availability of pathology service		1.869	1.863	.000	.993	2.746

Those who can read and write (from grade 1 to Master Degree, doesn’t mean all of them knowledgeable)

## Discussion

The satisfaction of caregivers was assessed in the pediatric ward of a large referral hospital in southwest Ethiopia. The magnitude of caregiver’s satisfaction in Jimma University Medical Center's pediatric wards was 68%. The result was higher than in Botswana, where caregiver satisfaction was 29.2% [10] and lower than in the United States of America, where caregivers satisfaction was 75% [20]. The difference might be attributed to socioeconomic differences between countries, which may contribute to differences in health systems. Our result is slightly higher than that of another study in the country, which exposed that parental satisfaction with the neonatal intensive care unit was 57.9%(95%CI: 49.1, 66.7) [8]. The difference might be due to participant differences that mean in intensive care unit clients are in the critical level and potential outcomes in the neonatal intensive care unit.

Level of education, basic hospital facilities, patience to listen to patients' problems, staff communication with caregivers, availability of laboratory tests and their service, availability of the drug, imaging, and service at radiology, and availability of pathology and its service are all factors that have a significant effect on caregiver satisfaction.

There is a link between education and satisfaction of customers. People who are more educated are happier and more satisfied than those who are less educated. In this study, Caregivers who could not read or write were 0.24 units less satisfied than literate caregivers. This could be because caregivers who have been educated are more informed about the service and are more likely to defend their rights. This finding is consistent with the findings of a Nepalese study [16]. In Nepal, educated caregivers were more satisfied with the service provided to their children than uneducated caregivers.

The cleanliness, comfort of the ward during their stay, the suitability of the ward for caregivers, and waiting times all have an impact on how satisfied the caregivers are in the pediatric ward. Caregivers were satisfied with the cleanliness and location of the wards; however the majorities were dissatisfied with the hospital's easy access to the emergency department and the length of time it took to enter the physician room. Caregivers were also dissatisfied with the ward's suitability/comfort during their stay. The study's findings were consistent with previous research conducted in low-income countries, where caregivers were dissatisfied with hospital settings [10, 27]. Many caregivers in developing countries did not believe that the instruments were clean and up to date. The findings from Botswana's studies also show that caregivers were dissatisfied with the hospital's structure, ease of finding services point, and wait time [10].

In Ethiopia, basic amenities such as water supply, electricity, bathrooms, communication equipment, and sanitation facilities in health facilities are critical [28]. The satisfaction of caregivers and patients depend on the availability of these fundamental amenities in healthcare facilities. In this study, more than half of study participants were unhappy with these basic amenities. The caregivers believed the pediatric ward was inappropriate for them, and the food service was inconvenient. They were also dissatisfied with the availability of water, the bathroom, its cleanliness, and its accessibility. This finding is consistent with the findings of a Botswana study [10]. Caregiver satisfaction was directly related to the availability of laboratory tests. This finding is consistent with other studies conducted in the country [29]. According to the Donabedian model, which is linked by three domains of structure, process, and outcome, there is a strong relationship between the availability of inputs such as basic facilities and the outcome of the service.

Personalized service, integrity, empathy, and staff willingness to invest time and effort in patients' wellness all influence patient experience and satisfaction [30]. Caregivers in this study were dissatisfied with the availability of assigned nurses and doctors when they were needed. The findings were consistent with those of a previous hospital study, which found that nearly half of caregivers (47.3%) do not believe doctors or nurses in charge will take responsibility for their child [8]. Similarly, caregivers in this study were dissatisfied with how nurses communicated with them and with the respect shown by supportive staff. Caregivers' satisfaction increased when patients received clear communication from providers. Good communication increases patient satisfaction, improves health outcomes, and strengthens treatment adherence [31]. In our study, only about 15% of caregivers were satisfied with the communication. Other findings give support to the conclusion. Caregivers in Nepal [16] were dissatisfied with how health professionals communicated with them during treatment; providers did not communicate with or discuss treatment with the parents.

According to this study, caregiver satisfaction is directly correlated with how attentively healthcare professionals listen to their patients' complaints. Compassionate communication during consultation is a necessary prerequisite for the delivery of high-quality health care services and the effective achievement of patient-centeredness [12, 15, 17]. This study's findings are comparable to those of a study conducted in Jimma Medical Center's neonatal intensive care unit and in Botswana [8, 32]. This study is limited to patients who visited our facility; a more powerful study design would be used to assess the level of satisfaction of other caregivers. Furthermore, it is critical to use this result to improve the weak point of pediatric service.

### Limitation

Although the study used standard tools to assess caregiver satisfaction, it had limitations, including the possibility of caregivers under or over reporting their level of satisfaction. Furthermore, because the data is cross-sectional, it is difficult to draw a causal effect relationship. Despite this limitation, we believe that our study has very important findings for improving pediatric hospital services in the study area and other areas with similar setups.

## Conclusion

In conclusion, caregivers were moderately satisfied. Education level, availability of basic facilities such as piped water, a bathroom, and a latrine; communication, and the availability of laboratory tests and drugs were all factors that significantly associated with caregiver satisfaction. It is thus recommended that the hospital should avail laboratory tests and drugs in order to maintain high levels of caregiver satisfaction. Moreover, the communication between health professionals and the patient should be improved. In addition, since the overall objective of the health professionals is to provide quality care that meets caregivers' needs and promotes their satisfaction; therefore they must understand and be patient with them.

## Data Availability

The data described in this article can be freely obtained from the first author or corresponding author on these emails: mohammedbeshir84@yahoo.com, tilahunfufa@gmail.com.
